# Preparation Method of Upconversion Nanoparticles and Its Biological Application

**DOI:** 10.3390/nano16020148

**Published:** 2026-01-22

**Authors:** Liang Li, Ming Li

**Affiliations:** School of Medicine and Health, Yancheng Polytechnic College, Yancheng 224005, China

**Keywords:** upconversion nanoparticles, synthesis method, biological application

## Abstract

Next-generation therapeutic devices will rely on an intelligent integrated system that consolidates multiple functions into a single platform. These individual chemical components exhibit diverse physicochemical properties, demonstrating multifunctional characteristics. In this review, we focus on how the distinctive properties of upconversion nanoparticles (UCNPs), achieved via refined preparation methods, unlock novel functionalities in biomedical applications. Specifically, features such as near-infrared excitation, deep-tissue penetration, low autofluorescence, and tunable multicolor emission endow UCNPs with substantial potential in fields including deep-tissue imaging, targeted drug delivery, and photodynamic therapy. This article systematically reviews recent advances in the design and functionalization of UCNPs, elucidating their role in facilitating the development of integrated diagnostic and therapeutic platforms and fostering the establishment of intelligent responsive treatment systems. Finally, we address current technical challenges—including uniformity in large-scale production, long-term biosafety, and in vivo metabolic mechanisms—and provide insights into future interdisciplinary integration, clinical translation pathways, and their potential role in personalized medicine.

## 1. Introduction

Nanoscience and technology have advanced rapidly in the past decade. Recently, scientists’ attention has been increasingly focused on the application of nanomaterials in biology, and as a new generation of light-emitting materials, upconversion nanoparticles (UCNPs) have caught their wide attention [1,2,3]. In the past decade, the development of upconversion nanoluminescent materials has promoted the transformation of fluorescence imaging from micro to macro. Although other common fluorescent materials (including organic dyes, fluorescent proteins, metal complexes or semiconductor quantum dots) have made significant progress in real-time detection and bioimaging as basic biomarkers [4,5], they still have some drawbacks. These fluorescent materials often need to be excited by ultraviolet (UV) or visible light, which can induce spontaneous fluorescence of biological tissues, DNA damage and cell death of biological samples, resulting in low signal-to-noise ratio and limited sensitivity [4,5,6]. In addition, the broad spectrum of emission spectra of these fluorescent materials makes them unsuitable for multiple biomarkers and have poor light stability.

In contrast, UCNPs have many good properties. The main difference between UCNPs and other light-emitting imaging materials is that they can emit visible or ultraviolet light when exposed to near-infrared light. Non-radiation does not cause photodamage to biological tissues, and can effectively avoid self-fluorescence of organisms, with high detection sensitivity and high light penetration depth [7,8]. In addition, UCNPs also show the advantages of narrow emission bandwidth, high light stability, controllable emission spectrum, long life and low cytotoxicity [2,9,10]. Although there are already numerous reviews on UCNPs, this review aims to offer a practical guidance framework for researchers in the biomedical field, focusing on how to select the most appropriate synthesis methods and surface modification strategies based on specific biological application scenarios (such as deep-tissue imaging, in vitro diagnosis, etc.), and analyzing the key challenges and future opportunities faced by this field in transitioning from basic research to clinical translation.

## 2. Luminescence Mechanism and Composition of Upconversion Nanomaterials

### 2.1. Composition of Upconversion Nanomaterials

Rare-earth (RE)-doped upconversion nanoparticles (UCNPs) are defined by their unique ability to convert low-energy excitation photons into higher-energy emission photons, a process known as photon upconversion, which has recently received considerable attention. In general, rare-earth-doped UCNPs consist of three components: a master matrix, a sensitizer and an activator. The main matrix is one of the most important components of upconversion nanoparticles, because they can provide a suitable crystal field for the luminescence center and fix the doped ions. In the sensitized luminescence process, the sensitizer can be efficiently excited by the energy of the incident light source and transfer this energy to the activator that can emit radiation through radiation-free relaxation, making it transition to a higher energy level and emit upconversion fluorescence. Thus, the activator is the luminescent center of the upconversion nanoparticle, while the sensitizer enhances the upconversion luminescence efficiency. Generally, dopants, i.e., sensitizers and activators, are added to the main matrix in relatively low concentrations (usually about 20% for sensitizers and <2% for activators).

Inorganic compounds of trivalent rare-earth ions are usually ideal as the main matrix for upconversion nanoparticles. However, a principal matrix with low lattice photon energy requires minimizing non-radiative losses and maximizing upconversion emission. Trivalent Yb3+ ions with simple energy level structures are suitable for use as upconversion sensitizers [11]. A huge majority of rare-earth ions with three positive charges have many excited levels and are suitable for use as upconversion activators [12,13,14].

### 2.2. Luminescence Mechanism of Nanomaterials

Upconversion is a nonlinear optical process, which refers to the process in which a material absorbs two or more low-energy (near-infrared light) photons and emits high-energy (visible or ultraviolet light) photons, so it can be classified as the anti-Stokes mechanism. The upconversion luminescence mechanism can be summarized into three main types: excited state absorption (ESA), energy transfer (ET) and photon avalanche (PA) [15,16].

**Figure 1:**
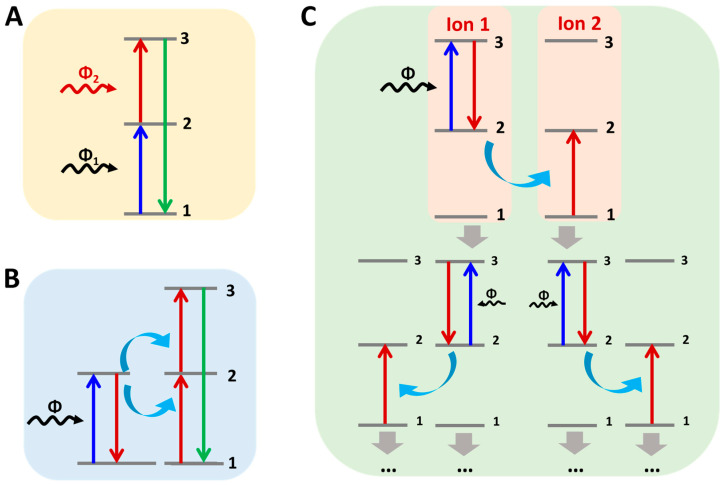
Luminescence mechanism diagram of nanomaterials: excited state absorption (**A**), energy transfer (**B**) and photon avalanche (**C**).

(1) Excited state absorption: Excited state absorption, also known as continuous two-photon absorption, is one of the most widely recognized models of upconversion luminescence [17]. Excited state absorption refers to the process of continuous absorption of multiple photons by an ion from a low-energy ground state level to a high-energy excited state level, and upconversion luminescence is generated when it returns to the ground state. The excited state absorption (ESA) method involves an ion sequentially absorbing two photons, with the second absorption occurring from an already occupied excited state. As shown in Figure 1A, under a suitable excitation light source the first photon causes ions to enter the metastable intermediate excited state (state 2) from the ground state (state 1), which is called ground state absorption (GSA). If the energy difference between the intermediate excited state (state 2) and the higher excited state (state 3) matches the energy of the excitation light source, the ion will continue to absorb the second photon from state 2 to the higher excited state (state 3), and when the ion returns from the higher excited state (state 3) to the ground state (state 1), the upconversion luminescence will occur.

(2) Energy transfer: Energy transfer involves two or more ions. Unlike the excited state absorption process, energy transfer process 1 involves the exciting ion (sensitizer or donor) absorbing the energy provided by the light source and transferring the energy to another adjacent ion (activator or acceptor). Different types of ET mechanisms [18,19] that are well known include continuous energy transfer (SET), cross relaxation (CR), cooperative sensitization (CS), and cooperative luminescence (CL). For example, the energy transfer process of SET is shown in Figure 1B; an activated ion in state 1 is elevated to state 2 by ET. The activated ion is then promoted again to state 3 by a second ET. Only the sensitized ions can absorb photons from the incident light in this process.
(3) Photon avalanche: Also known as absorption avalanche, photon avalanche was first discovered by Chivian and is one of the most efficient types of upconversion [18]. Of all the upconversion processes, the photon avalanche process is the least observed. Figure 1C shows the simple energy transfer process of the photon avalanche process. Initially, the sensitized ion (ion 1) in state 1 is promoted to state 2 by GSA. Next, an incident photon is pushed into state 3 by ESA. The basic process of photon avalanche is that a sensitized ion in state 3 (ion 1) can interact with an adjacent ion in the ground state (ion 2), and as a result of cross relaxation two ions are produced in state 2 (ion 1 and 2). Two newly created ions acting as sensitized ions can produce an additional four ions, which in turn can produce another eight ions, and so on. Finally, the intermediate excited state (state 2) acts as a storage vessel for energy and can build up an avalanche of ions in state 2.

## 3. Synthesis Method of Upconversion Nanoparticles

### 3.1. Thermal Decomposition Method

**Figure 2:**
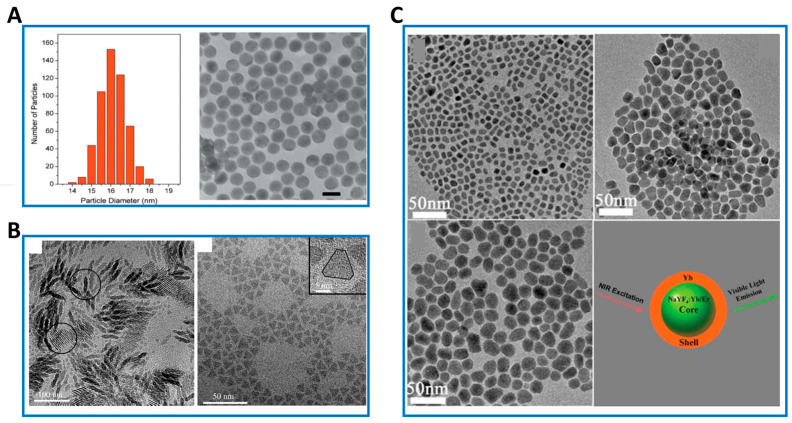
(**A**) Histogram of the NaGdF_4_:Er^3+^, Yb^3+^ active-core/NaGdF_4_:Yb^3+^ active-shell nanoparticle size distribution (>500 nanoparticles) obtained from TEM. Average particle size = 15.9 ± 0.7 nm (left panel) and TEM image of NaGdF_4_:Er^3+^, Yb^3+^/NaGdF4:Yb^3+^ (active-core/active-shell) nanoparticles. Scale bar = 20 nm. (right panel) (Reprinted with permission from Ref. [22]. Copyright 2009 John Wiley and Sons). (**B**) TEM image of GdF_3_ zigzag-shaped nanoplates (left panel) and LaF_3_ nanoplates synthesized at 300 °C with OA/ODE = 1:1 over 30 min (right panel) (Reprinted with permission from Ref. [25]. Copyright 2007 John Wiley and Sons). (**C**) TEM images of the core-only and core–shell architectured NPs and the corresponding schematic image. Upleft panel: The core-only NPs (~10 nm), Upright panel: BGF@Yb NPs (~15 nm), Left bottom panel: BGF@Yb (~19 nm), Right bottom panel: the schematic image of the core–shell structure (Reprinted with permission from Ref. [23]. Copyright 2011 Royal Society of Chemistry).

Thermal decomposition method refers to the method of thermal decomposition of inorganic or organic precursors in high boiling point organic solvents, and is the traditional method used to prepare inorganic nanocrystals [19,20,21,22]. The specific process is generally composed of the following parts: (1) At room temperature, a certain amount of RE (CF_3_COO)_3_ precursor is added to the mixture of oleic acid (OA), 1-octadecene (OD) and organic oil amine (OM); (2) under the protection of argon and intense magnetic stirring, the solution is heated to 165 °C for 30 min to remove water and oxygen; (3) Under the protection of argon, the solution is heated to a high temperature (usually greater than 300 °C) for a period of time, and then the nanocrystals are cooled to room temperature to collect the products from the reaction mixture. The reaction temperature, reaction time, and the molar ratio of OA, OD, and OM in the reaction mixture all have an effect on the final nanocrystals. It should be noted that OM is sometimes introduced as a necessary component to regulate the reaction environment so that final products with different forms and sizes can be obtained (Figure 2).

The most outstanding advantages of this method are the high quality of the product, the pure crystal phase and the strong upconversion luminescence ability [23,24]. However, this method also has the following disadvantages: (1) Pre-synthesis of RE (CF_3_COO)_3_ precursors is usually required; (2) the decomposition of trifluoroacetate produces toxic fluorinated and oxyfluorocarbon substances at the same time, so the reaction needs to be carefully handled in the fume hood; (3) the reaction environment requires an inert atmosphere and dehydration, which further increases the difficulty of operation. In the pyrolysis method, the key factor to achieve size controllable and monodisperse UCNPs is the need to select the appropriate ligand. Yan et al. report that oleoamine ligands have significant effects on fluoride ions and are an important buffer, because as the RE series atomic number increases, the alkalinity of RE oxides gradually decreases, so the lighter the rare-earth element, the more OM is required [25].

### 3.2. Coprecipitation Method

**Figure 3:**
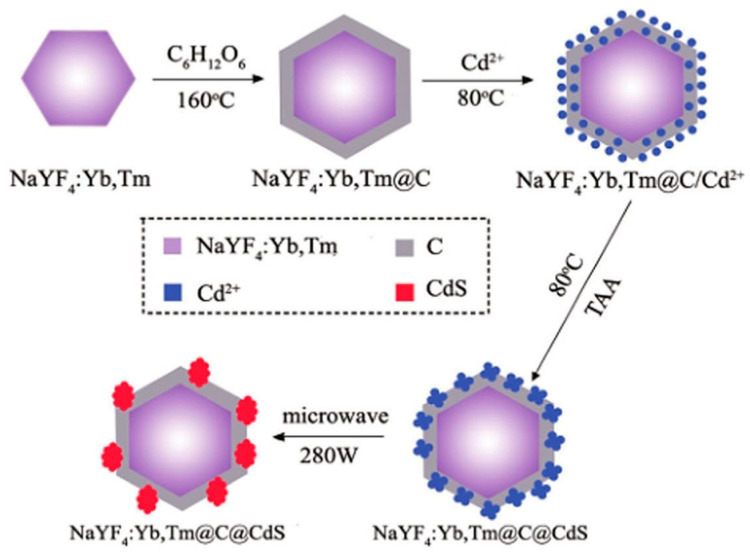
The coprecipitation method to synthesize the core/shell nanoparticles of NaYF_4_:Yb,Tm@C@CdS (Reprinted with permission from Ref. [26]. Copyright 2019 John Wiley and Sons).

Due to the limitations of the thermal decomposition method, the coprecipitation method was developed and has been used as one of the most convenient methods for UCNPs synthesis [26,27]. The experimental process generally consists of the following parts: (1) RE salt is mixed with a solution of OA, OD (sometimes added with OM) at a certain proportion, heated to 165 °C and kept for 30 min, and then cooled to room temperature; (2) Methanol solution of NH_4_F and MOH (M = Li, Na, K) is added to the mixture and stirred for 30 min; (3) After removing the methanol and residual water by evaporation, the reaction mixture is heated to a high temperature (usually >300 °C) under the protection of argon to produce the desired nanocrystals (Figure 3). The advantages of the co-precipitation method are simple operation, non-toxic byproducts and low cost, so it is widely used in the preparation of various materials [28,29].

Although the synthesis of UCNPs by the co-precipitation method has been significantly improved and has universal practicability, it is troubled by the long continuous operation of the experimental process, which usually takes more than 5 h, including the removal of methanol solvent and the water generated during the synthesis process, and controlled crystal growth at a certain high temperature. In addition, the largescale synthesis of UCNPs using this method remains a major challenge.

### 3.3. Hydrothermal/Solvothermal Method

The hydrothermal/solvothermal method is usually enacted through a chemical reaction process between positive and negative ions, under high temperature and high pressure conditions to precipitate the product from the solvent, after appropriate treatment in the solvent to obtain nanoscale materials [30,31,32,33]. Because the method is simple to operate, the experimental process does not require strict operation, and the reaction temperature of the hydrothermal/solvothermal method is usually lower than that of the thermal decomposition method and co-precipitation method, it is now widely used in the process of synthesizing UCNPs [34,35,36,37,38]. The advantages of using the hydrothermal/solvothermal method to synthesize high-quality UCNPs include: (1) high product purity, (2) easy control of the size, structure and morphology of the nanoparticles, (3) relatively low reaction temperatures (generally below 200 °C), and (4) the use of simple equipment and a simple overall process. However, in most cases, the hydrophilicity of UCNPs prepared using the hydrothermal/solvothermal method is insufficient due to the presence of hydrophobic organic ligands (such as OA) on the nanoparticle surface. Therefore, in order to improve the water solubility and biocompatibility of UCNPs, surface modification is a top priority (Table 1).

### 3.4. Sol–Gel Method

**Figure 4:**
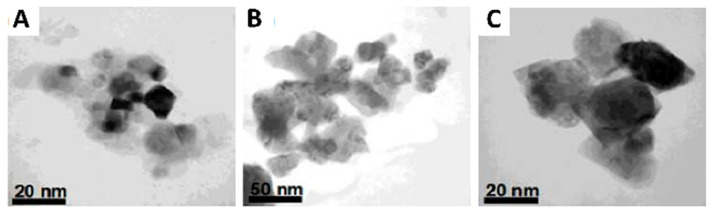
TEM images of the 1.0 mol% Er^3+^-doped BaTiO_3_ nanoparticles obtained after heating to three different temperatures: (**A**) 700 °C; (**B**) 850 °C; and (**C**) 1000 °C (Reprinted from Ref. [39]).

Sol–gel method is a typical wet chemical method for the synthesis of UCNPs [39,40,41,42], which can be generally divided into three types: (1) sol–gel route based on the hydrolysis and condensation of molecular precursors [43]; (2) gelation route based on condensation of aqueous solution containing metal chelates; And (3) polymerizable complex routes. In the sol–gel method, rare-earth nitrates or metallic alkols are usually used as starting reactants. By mixing the reactants in the liquid phase, a hydrolysis and condensation reaction is initiated, followed by a period of annealing at high temperatures [44]. For the sol–gel method, the annealing process (temperature and time) is a critical step in the preparation process and can seriously affect the quality of the sample (Figure 4). It should be noted that although the sol–gel method can be used for large-scale production, and the high crystallinity of the product formed at high annealing temperatures can provide high luminous intensity, the sol–gel derived nanocrystals usually have a wide particle size, irregular distribution, irregular morphology, and are insoluble in water, which are the disadvantages of the method.

## 4. Surface Modification of Upconversion Nanoparticles

Due to the effects of impurities and lattice defects on synthesized UCNPs, the quantum efficiency of UCNPs is lower than that of the corresponding bulk materials. In addition, because UCNPs are usually prepared in an organic environment, the particle surface is surrounded by hydrophobic molecules [39,45,46,47], so they are mostly insoluble in water. Therefore, it is important to develop appropriate schemes to make them hydrophilic while maintaining upconversion efficiency to meet various requirements. For example, an ideal luminescent nanocrystal with good biocompatibility should meet several requirements, including (1) high luminescent efficiency and low background noise; (2) good solubility and stability in the biological environment; (3) good biocompatibility, (4) appropriate size^1^ (less than 100 nm).

However, UCNPs weakness is low upconversion efficiency [48,49]. At present, the urgent task is to improve the upconversion efficiency. Introducing inert crystal shells of undoped materials around each nanocrystalline doped with rare earth ions is an effective choice to improve the luminous efficiency of UCNPs [50,51,52]. The shell material usually has the same composition as the core master crystal, which can effectively reduce surface fluorescence quenching. In such structures, all doped ions are confined to the core of the nanocrystals, effectively inhibiting the non-radiative energy transfer from rare earth ions to the surface quenched site, which results in an increase in upconversion luminescence efficiency. Wrapping 1.5 nm thick NaYF_4_ shells on 8 nm NaYF_4_:Yb,Tm nanocrystals enhanced their luminescence by nearly 30 times, as reported in [53,54].

### 4.1. SiO_2_ Coating Method

**Figure 5:**
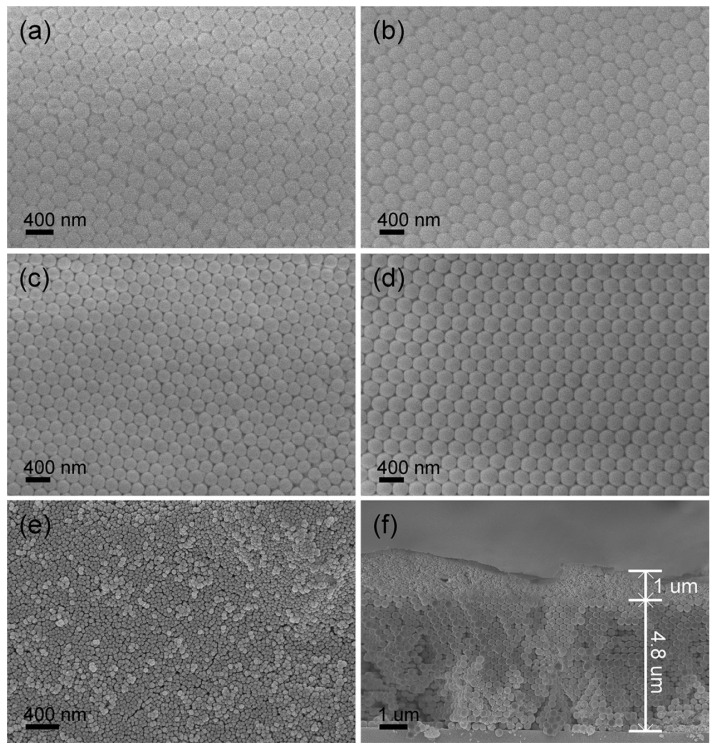
SEM images of SiO_2_ opal PCs: (**a**,**c**) top view of PCs assembled by 225 nm silica microspheres before and after calcination; (**b**,**d**) top view of PCs assembled by 270 nm silica microspheres before and after calcination; (**e**,**f**) top view and sectional view of PCs spin-coated with UCNPs (Reprinted with permission from Ref. [60]. Copyright 2019 Springer Nature).

SiO_2_ coating involves the growth of amorphous silica shells on UCNPs cores [55,56] because it is essentially achieved by chemically and physically pre-treating the surface of UCNPs to generate “active sites.” Under precisely controlled reaction kinetics, silicic acid oligomers are preferentially guided to undergo polycondensation at these sites, leading to heterogeneous nucleation and growth of a dense, amorphous silica network shell on the UCNP surface. Since SiO_2_-coated UCNPs are rich in amino and carboxyl groups, amino and carboxyl group functionalization can be further utilized to achieve biomolecular coupling and their application in tumor therapy (Figure 5) [57,58]. It provides a platform for the multifunctionalization of UCNPs. The disadvantage is that the method is time consuming and difficult to synthesize on a large scale. In addition, the coated SiO_2_ layer may also affect the luminous intensity of UCNPs through light scattering, because the upconversion efficiency of UNCPs is already very low, so it cannot be an ideal surface modification method. Therefore, there is still a lot of work to be done to improve the general applicability of this method.

### 4.2. Polymer Coating Method

**Figure 6:**
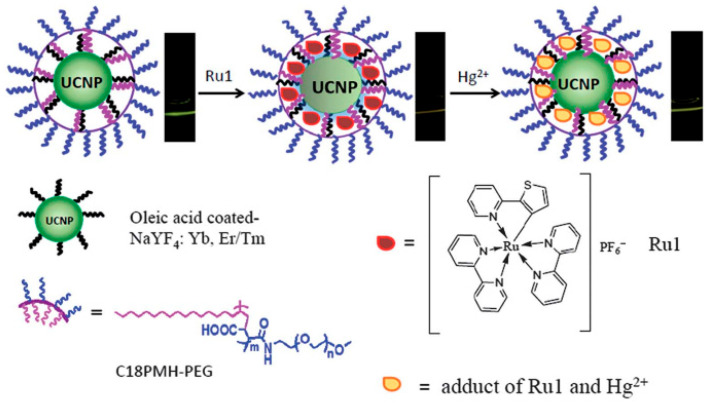
Schematic illustration of the synthesis of a UCNP-based nanosystem and its application in upconversion luminescence detection of Hg^2+^ (Reprinted with permission from Ref. [65]. Copyright 2009 Royal Society of Chemistry).

The polymer coating method refers to the use of the van der Waals effect between the hydrophobic chain of the amphiphilic polymer molecule and the hydrophobic chain on the surface of the nanoparticle [59,60,61,62], so that the polymer molecule is coated on the surface of the nanoparticle, and its hydrophilic end is exposed to make the nanoparticle water soluble. The polymer coating of UCNPs is not a simple “wrapping” process, but rather a dynamic self-assembly process driven by molecular design at the nano-interface. By precisely utilizing hydrophobic interactions as the “lock” and constructing a protective hydrated corona via hydrophilic segments, this approach achieves the stable phase transfer of UCNPs from the organic to the aqueous phase [63,64]. Furthermore, it provides a highly designable and flexible interfacial platform for subsequent biofunctionalization. While its major advantages lie in operational simplicity and excellent biocompatibility, the core challenge remains in enhancing the coating robustness and long-term stability. The multifunctionalization of upconversion nanoparticles can also be achieved by the amphiphilic coating. UCNPs can be formed by coating an amphiphilic polymer (C_18_PMH-PEG) on NaYF_4_:Yb/Er (Figure 6) [65], Tm nanoparticles based on hydrophobic–hydrophobic interactions [66,67,68]. Therefore, the primary advantages of the polymer coating method lie in its operational simplicity and excellent biocompatibility, while the core challenge remains how to enhance the binding strength and long-term stability of the coating layer. To further advance this technology, research efforts should focus on two key directions: strengthening interfacial binding strategies and developing stability-enhancement mechanisms.

### 4.3. Ligand Oxidation Method

The ligand oxidation method produces hydrophilic functional groups by oxidizing unsaturated carbon–carbon bonds contained in ligands on the surface of upconverted nanoparticles [69,70,71,72]. It is reported that a large number of carboxylic acid groups will be produced on the surface of UCNPs after oxidation [73], which not only makes the nanocrystals have good solubility in water, but also can be directly chemically coupled with biomolecules. The oxidation process has no significant negative effects on the shape, crystal structure, chemical composition and luminescence properties of upconverted nanomaterials [73,74]. However, this method is limited to those nanoparticles that contain unsaturated carbon–carbon bond ligands. Because the experimental conditions are very demanding, the oxidation process may also result in the ligands being eliminated directly. If the oxidation of ligands can be achieved under mild experimental conditions, there will be no or less damage to the surface of UCNPs, which would be a more ideal situation. In addition, prolonged oxidation may result in the formation of brown MnO_2_ by products, which are not easily separated and will weaken the upconversion fluorescence intensity [75,76] (Table 2).

## 5. Biological Applications of Upconversion Nanoparticles

### 5.1. In Vitro Cell Imaging and in Vivo Tissue Imaging

Excitation of rare-earth-doped UCNPs usually requires only IR radiation. The use of IR radiation is of great advantage in cell and tissue imaging because they provide (1) a high signal-to-noise ratio; (2) long wavelength excitation light with great penetration (up to several inches of tissue penetration); and (3) long-term irradiation causes less light damage to cells/tissues [77,78]. Therefore, rare-earth-doped UCNPs are a good alternative to traditional fluorescent biomarkers (such as organic dyes and quantum dots) for in vitro cell imaging and in vivo tissue imaging.

**Figure 7:**
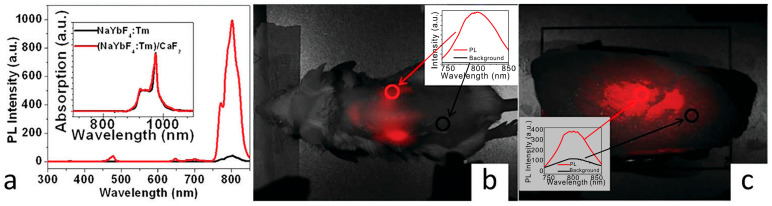
(**a**) Upconversion photoluminescence spectra of core (NaYF_4_:Tm) and core–shell (NaYF_4_:Tm@CaF_2_) UCNPs under NIR illumination. (**b**) Imaging of a BALB/c mouse injected with the hyaluronic acid modified core–shell UCNPs through tail vein injection. (**c**) Photoluminescent image of the cuvette containing core–shell UCNPs covered with a piece of 3.2 cm pork tissue (Reprinted with permission from Ref. [77]. Copyright 2018 Royal Society of Chemistry).

(1) Labeling and imaging of in vitro cells. The application of UPCNs in in vitro cell imaging can be divided into two scenarios. One involves the covalent conjugation of targeting ligands (e.g., folate, aptamers, proteins, or polypeptides) onto the surface of UCNPs. Such surface modifications enable targeted in vitro cellular imaging and ligand-directed delivery of tracer molecules. The other utilizes cellular endocytosis to internalize UCNPs, followed by fluorescence imaging to investigate the mechanisms and pathways of cellular uptake. Chatterjee et al. [79] used NaYF_4_:Yb, Er UCNPs for cell imaging for the first time, which were functionalized by PEI and then covalently combined with folic acid to form folate-modified NaYF_4_:Yb, Er UCNPs. Folate-modified NaYF_4_:Yb, Er UCNPs were then cultured with human HT29 breast cancer cell and human OVCAR3 ovarian cancer cells under physiological conditions for 24 h. UCNPs modified with folate were able to specifically target the cells because of unusually high levels of folate receptors expressed on the surfaces of both types of cells, and when the folate modified UCNPs was connected to the cells, UCNPs could glow green upconversion fluorescence under a confocal microscope equipped with a 980 nm laser (Figure 7).
(2) In vivo tissue imaging. Chatterjee et al. [79] first reported the use of upconversion nanoparticles for in vivo imaging of deep tissue in Wistar rats. In their study, NaYF_4_:Yb coated with PEI (5 wt %) and Er UCNPs were first used, and then 100 μL PEI-coated UCNPs (4.4 mg/mL) were injected subcutaneously into the groin and thigh sites of rats at a depth of 10 mm. The rats were then stimulated with a 980 nm excitation light source. The results showed that UCNPs injected into the skin of the abdomen, the muscle of the thigh and under the skin of the back showed visible fluorescence under a 980 nm excitation light source. However, when the quantum dots (used as a control) were injected into the thicker skin of the back or abdomen, they did not show any fluorescence under UV excitation, and only the quantum dots injected into the translucent skin of the feet fluoresced. Thus, NIR radiation has been demonstrated to have better penetration than UV light, and NIR light-stimulated UCNPs has great potential for in vivo imaging and their application in tumor therapeutics. However, judging from the existing literature, the use of UCNPs for in vivo imaging is still at a preliminary stage.

### 5.2. Biological Detection and Analysis

**Figure 8:**
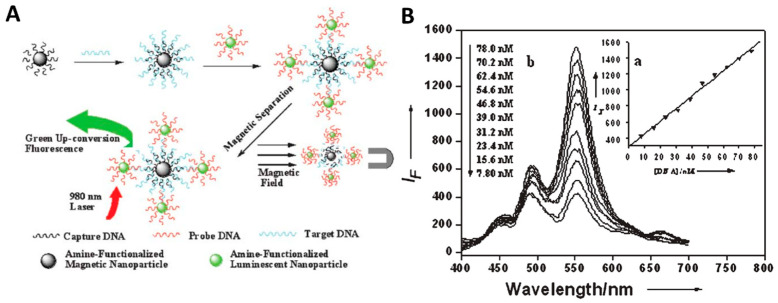
(**A**) Scheme of the DNA assay method combining UC fluorescence with magnetic separation. Magnetic nanoparticles were modified with capture DNA and phosphor nanoparticles were modified with probe DNA, respectively. Capture-DNA-modified magnetite nanoparticles were hybridized with target DNA and separated with an assistant magnetic field. The probe-DNA-modified luminescent nanoparticles were then conjugated to the magnetic nanoparticles through the hybridization with the overhanging region of the target sequences. The binary nanoparticles were purified with magnetic separation and detected with upconversion fluorescence technology. The excitation light was a commercially available 980 nm laser. (**B**) Fluorescence spectra of binary nanocomposite in the presence of different concentration of target DNA (b) and the linear relationship (a) between luminescence intensity and target DNA content according to (b) (Reprinted with permission from Ref. [86]. Copyright 2006 Royal Society of Chemistry).

(1) Flourescence Resonance Energy Transfer (FRET)-based detection. FRET is a process that occurs when energy is transferred between a donor and recipient. When the absorption spectra of two fluorescent molecules (energy donor and energy accepter) overlap in a certain region, and the distance between the donor and the accepter is less than 10 nanometers, the fluorescence molecule as an energy accepter can absorb the photon radiated by another fluorescent molecule as an energy donor, and energy transfer occurs. This process is called FRET. In recent years, FRET-based analytical methods have received considerable attention as an important tool for biological detection due to their ease of operation and high sensitivity.
(2) Direct detection of biomolecules, also known as heterophase detection, refers to the specific recognition and high affinity between the molecules to be measured fixed on the substrate and the rare-earth-doped UCNPs labeled probe molecules [80,81,82,83,84]. The detection based on bioaffinity system is realized according to the proportional relationship between the concentration of the object to be measured and the fluorescence intensity [85]. Wang et al. [86] used NaYF_4_:Yb, Er UCNPs to design a fluorescence sensor based on magnetic field separation to detect trace DNA sensitively (Figure 8). Firstly, Fe_3_O_4_ magnetic nanoparticles were covalently linked to the capture DNA, and NaYF_4_:Yb, Er UCNPs were covalently linked to the probe DNA. When the target DNA is added to the system, the long strand of target DNA can specifically recognize the complementary sequence fragments on the trapping DNA connected to the Fe_3_O_4_ magnetic nanoparticle and the probe DNA connected to the UCNPs, and finally form a three-strand nanocomplex, which is then purified by magnetic separation. It was found that the upconversion fluorescence intensity of UCNPs was linearly correlated with the concentration of target DNA in the range of 7.8–78.0 nm. This method is a good choice for detecting trace amounts of DNA.

### 5.3. Diagnosis and Treatment

The application of upconversion nanoparticles in the field of tumor diagnosis and treatment mainly involves the following three aspects:

(1) Drug delivery. The UCNPs-based drug delivery system can be used for drug tracers, evaluation of drug-delivery efficiency and study of drug-delivery mechanisms [87]. To date, most UCNPs drug-delivery systems have been coated with PEG [88,89] or mesoporous silica [90,91,92]. Liu and colleagues functionalized the amphiphilic polymer PEG by wrapping it in UCNPs, then loaded DOX molecules with pegylated UCNPs and covalently bound to FA for targeted drug delivery and cell imaging. The loading and release of DOX in UCNPs was controlled by changing pH; the drug dissociation rate increases in acidic environments, which is conducive to the control of drug release [93].
(2) Photodynamic therapy (PDT). PDT is a relatively new clinical treatment method, which refers to the photosensitized agent that converts the adsorbated oxygen into reactive oxygen species or singlet oxygen to kill disease cells under the condition of high-energy excitation light [94,95,96]. However, the high energy of the excitation light source is mostly visible light or even ultraviolet light, which limits the penetration depth of photodynamic therapy, and it is impossible to treat deep-seated tumors or too-large tumors [97]. While upconversion nanomaterials can emit visible light when excited by near-infrared light, UCNPs can be used to activate photosensitizers in deep tissues, and the penetration depth of tissues can be increased due to weak absorption in the optical “transparent window”.
(3) Photothermodynamic therapy (PTT). PTT is a treatment that absorbs radiation light energy through a light absorber to generate heat [98], resulting in the local temperature of tumor cells being too high and thus dying [99]. Various nanomaterials with high near-infrared absorbance, such as gold and silver nanoshells, nanorods and nanocages, have been used for PTT treatment of tumors [100]. Song et al. reported the hexagonal phase NaYF_4_ of the core–shell structure and their unique biological functional properties [101] showed that HepG2 cells from human liver cancer and BCap-37 cells from human breast cancer were cultured with UCNPs in vitro and photothermally induced death occurred when exposed to a 980 nm excitation light source.

### 5.4. Optogenetics

**Figure 9:**
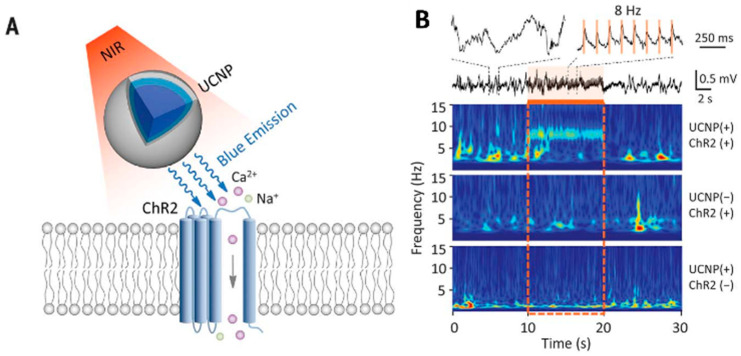
(**A**) Schematic principle of UCNP-mediated NIR upconversion optogenetics. (**B**) Hippocampal LFP in response to 8 Hz transcranial NIR stimulation (15 ms pulses, 10 s, 3.0 W peak power, 360 mW average power) of MS under different conditions. Top: Raw LFP trace from mouse with both UCNP and ChR2 injection. Bottom: Z-scored power in the theta range averaged across 30 s trials in all three conditions (Reprinted with permission from Ref. [104]. Copyright 2018 The American Association for the Advancement of Science).

Optogenetics has completely revolutionized the experimental study of neural circuits. It is expected to be used for treating neurological diseases [102]. However, it is limited because visible light cannot penetrate deeply into the brain tissue. UCNPs absorb near-infrared (NIR) light that penetrates the tissue and emit light of specific wavelengths [103]. UCNP technology will facilitate minimally invasive optical manipulation of neuronal activity, offering potential for remote therapy applications. A non-invasive deep brain stimulation technique based on UCNPs has been developed by Chen et al. [104] (Figure 9). The activation of dopamine neurons in the ventral tegmental area (VTA), the inhibition of hippocampal neurons, and the triggering of memory recall were successfully achieved. It demonstrated the application potential of UCNPs in various neural regulation scenarios. Hososhima et al. [105] combined Lanthanide element nanoparticles with optogenetics for the first time and verified the matching of Lanthanide element nanoparticles with photosensitive protein in vitro experiments, and achieved the activation of neurons, providing a new idea for deep brain stimulation. In addition, a NIR optogenetic system based on lanthanide nanoparticles (LNPs) and photosensitive proteins (such as ChR2) was also proposed [106]. The matching between LNPs and ChR2 was verified in in vitro experiments, and the activation of neurons was achieved, providing a new idea for deep brain stimulation. It can be seen from this that upconversion nanoparticles have provided an important technological breakthrough for deep brain stimulation and neural regulation, demonstrating the broad prospects of the combination of optogenetics and nanotechnology. In the future, more precise targeted delivery methods should be developed to reduce side effects and explore their application potential in more neurological disease models (Table 3).

UCNPs hold promise in biomedicine, but their clinical translation faces major hurdles in safety and biodistribution. A key concern is in vivo toxicity, driven by ion release (e.g., Y^3+^, F^−^) from dissolving matrices like NaYF_4_, which disrupts cell function. Toxicity is further influenced by particle size (smaller UCNPs are often more toxic) and coating stability; unstable coatings can expose hydrophobic cores, increasing cytotoxicity [107]. Following intravenous injection, UCNPs primarily accumulate in the liver and spleen via the mononuclear phagocyte system. Their clearance route is size dependent; larger particles (>10 nm) are slowly processed via the hepatobiliary system, while very small ones (<6 nm) can be rapidly cleared by the kidneys. The long-term fate of UCNPs involves gradual “biosolubilization” in biological fluids rather than conventional degradation. This dissolution rate is highly variable and dependent on the surface coating, with some polymers (e.g., PEG, PMA-g-dodecyl) offering significant protection. The ultimate metabolic fate and long-term effects of the released ions remain poorly understood. Overcoming these challenges requires rational surface engineering to control stability, targeting, and degradation, paired with comprehensive long-term safety studies to enable clinical advancement.

## 6. Conclusions and Perspectives

Upconversion materials possess the ability to convert near-infrared radiation with lower energy into visible radiation with higher energy through nonlinear optical processes. Due to their unique luminescent properties, upconversion luminescent substances exhibit numerous advantages, such as large Stokes shift, strong photostability, good chemical and physical stability, and low toxicity; near-infrared light excitation can reduce tissue damage to the organism while enhancing tissue penetration depth; and zero background in the imaging process, which can significantly improve detection and imaging sensitivity. Therefore, upconversion nanoluminescent materials have great development potential in biology.

The core challenge in the application of upconverting nanoparticles (UCNPs) in biological analysis lies in their low luminescence efficiency, which limits the detection sensitivity. The complex surface chemistry makes biological functionalization difficult and prone to stability issues. The uncontrollability of size and morphology affects their biological distribution and pharmacokinetics, and the long-term biological safety and in vivo metabolic pathways remain unclear. To address these problems, future development will focus on fundamentally improving the luminescence efficiency by designing new core–shell structures (such as sensitizing shells or heterojunctions) and exploring non-terrestrial materials; developing standardized and replicable biological coupling strategies to achieve stable multifunctionality; and using artificial-intelligence-assisted design to optimize synthesis parameters and performance. The ultimate goal is to achieve integrated multimodal diagnosis and treatment in deep tissues excited in the NIR-II region, combining high-resolution imaging with photodynamic/photothermal/chemotherapy, and promoting breakthrough applications in clinical translation areas such as high-sensitivity multiple detection, intraoperative navigation, and real-time-feedback-based drug monitoring.

## Figures and Tables

**Table 1:** Comparison of Major Synthesis Methods for UCNPs.

Method	Core Principle	Advantage	Disadvantage
Thermal Decomposition	Thermal decomposition of precursors (e.g., RE(CF_3_COO)_3_) in high-boiling-point organic solvents.	1. High product quality (pure crystalline phase, good monodispersity). 2. Strong luminescence intensity. 3. Morphology and size can be finely tuned via ligands (OA/OM).	1. Complex process; requires pre-synthesis of precursors, strictly anhydrous/oxygen-free conditions. 2. Toxic byproducts; decomposition of trifluoroacetates produces toxic fluorinated compounds. 3. High cost; requires high temperature and inert gas protection.
Coprecipitation	Co-precipitation of rare-earth salts and fluoride sources in an organic phase at high temperature to form nanocrystals.	1. Relatively simple operation. 2. Lower cost. 3. Non-toxic byproducts.	1. Time-consuming (typically >5 h). 2. Difficult to scale up. 3. Less precise control over crystal growth compared to thermal decomposition.
Hydrothermal/Solvothermal	Precipitation of products from solvent via ionic reactions under high temperature and pressure in water or organic solvents.	1. Simple equipment and operation. 2. Lower reaction temperature (typically <200 °C). 3. Easy control over particle size and morphology. 4. High product purity.	Products are typically coated with hydrophobic ligands (e.g., OA), resulting in poor water solubility and requiring further surface modification.
Sol–Gel	Formation of a sol via hydrolysis and condensation of precursors, followed by gelation and high-temperature annealing to obtain nanocrystals.	1. Suitable for large-scale production. 2. High crystallinity and strong luminescence after high-temperature annealing.	1. Broad particle size distribution and irregular morphology. 2. Poor water solubility. 3. Annealing process critically affects product quality and is difficult to control.

**Table 2:** Comparison of Major Surface Modification Methods for UCNPs.

Method	Core Principle	Advantage	Disadvantage	Primary Purpose
SiO_2_ Coating	Growth of an amorphous silica shell on UCNP surface via a sol–gel process.	1. Provides abundant surface functional groups (-OH, -NH_2_, -COOH) for easy multifunctionalization. 2. Effectively protects the core from chemical erosion. 3. Good biocompatibility.	1. Time-consuming process, difficult to scale up. 2. Silica shell may cause light scattering, reducing luminescence intensity. 3. The coating layer is relatively thick, which may affect performance.	Construction of multifunctional bioprobes and drug delivery platforms.
Polymer Coating	Physical coating via hydrophobic interactions between the hydrophobic chains of amphiphilic polymers and the hydrophobic ligands on the UCNP surface.	1. Simple and rapid operation. 2. Hydrophilic chains (e.g., PEG) provide excellent water solubility and biocompatibility. 3. Easy functionalization via polymer termini.	Coating relies mainly on hydrophobic interactions, resulting in insufficient long-term stability; may dissociate in complex environments.	Rapid achievement of water solubility for applications in biosensing and instant detection.
Ligand Oxidation	Oxidation of unsaturated carbon–carbon bonds in the native ligands on UCNPs (e.g., oleic acid) to generate hydrophilic groups (e.g., carboxyl groups).	Oxidation of unsaturated carbon–carbon bonds in the native ligands on UCNPs (e.g., oleic acid) to generate hydrophilic groups (e.g., carboxyl groups).	1. Limited applicability, only suitable for ligands containing unsaturated bonds. 2. Harsh conditions; over-oxidation may lead to ligand detachment or generation of hard-to-remove byproducts (e.g., MnO_2_), which quench fluorescence.	Direct hydrophilization and conjugation for specific systems with compatible surface ligand structures.

**Table 3:** Biological applications of UCNPs.

Application Fields	Advantages	Specific Applications	Research Stage/Challenges
In vitro cell imaging	Near-infrared excitation, high signal-to-noise ratio—low light damage	Targeted imaging: Surface-modified targeted molecules (such as folic acid) specifically mark the endocytosis mechanism of cancer cells. Research on the endocytosis pathway by cells taking up UCNPs	The technology is relatively mature.
In vivo tissue imaging	Near-infrared light has deep tissue penetration (up to several centimeters)—its penetration is significantly enhanced compared to ultraviolet light	When UCNPs is subcutaneously or intramuscularly injected into animal models (such as rats), fluorescence can be observed under 980 nm excitation. Traditional quantum dots cannot form images at the same depth	In the initial stage, further optimization of imaging depth and resolution is required
Biological detection and analysis	High sensitivity—Easy to operate	FRET detection: As an energy donor, a biosensor is constructed for direct detection; combined with magnetic separation technology, trace DNA is detected (linear range: 7.8–78.0 nm).	There is a considerable amount of methodological research, and it is in the process of being transformed into actual sample testing
Integrated diagnosis and treatment of tumors	Diagnosis; Image-guided therapy; Controlled release, deep therapy	Drug delivery: PEG/ mesoporous silica coating, ph-responsive release (such as DoX-loaded). Photodynamic therapy: activation of deep tissue photosensitizers. Photothermal therapy: NIR photothermal killing of tumor cells	Preclinical research is active, but the safety and targeting efficiency need to be optimized
optogenetics	Achieve non-invasive/minimally invasive deep brain region stimulation—near-infrared penetrates the skull, and UCNPs are converted into visible light to activate neurons	Successfully activating dopamine neurons, inhibiting hippocampal neurons, and triggering memory recall in animal models; verification of UCNPs matching with photopigments (such as ChR2)	In the frontier exploration stage, the accuracy and long-term safety of targeted delivery remain to be studied

## Data Availability

No new data were created or analyzed in this study. Data sharing is not applicable to this article.
